# Pulsed Field Ablation for Atrial Fibrillation: Mechanisms, Advantages, and Limitations

**DOI:** 10.31083/j.rcm2504138

**Published:** 2024-04-08

**Authors:** Shali Jiang, Frank Qian, Shuting Ji, Luohong Li, Qiming Liu, Shenghua Zhou, Yichao Xiao

**Affiliations:** ^1^Department of Cardiovascular Medicine, Second Xiangya Hospital, Central South University, 410011 Changsha, Hunan, China; ^2^Xiangya School of Medicine, Central South University, 410013 Changsha, Hunan, China; ^3^Department of Medicine, Beth Israel Deaconess Medical Center, Harvard Medical School, Boston, MA 02215, USA

**Keywords:** pulsed electric field, atrial fibrillation, catheter ablation, pulmonary vein isolation, irreversible electroporation

## Abstract

Pulsed field ablation with irreversible electroporation for the treatment of atrial fibrillation involves tissue-specific and non-thermal energy-induced cell necrosis, which helps avoid complications, such as pulmonary vein stenosis, atrial collateral tissue damage, and extensive atrial structural damage, often encountered with traditional thermal ablation. In existing clinical trials, pulsed field ablation has shown excellent effects on pulmonary vein isolation in patients with paroxysmal and persistent atrial fibrillation. Pulsed field ablation is easy, simple, and quick and can reduce iatrogenic injury. Therefore, the application of pulsed field ablation technology in the treatment of atrial fibrillation has a promising future. Notably, the adjustment of parameters in pulsed field ablation with different ablation catheter systems can strongly affect the area and depth of the necrotic myocardium, which greatly affects the likelihood of atrial fibrillation recurrence and incidence of adverse complications after ablation. In this paper, we review the mechanisms, advantages, and limitations of pulsed field ablation based on the results of a series of previous studies and provide ideas and directions for future research.

## 1. Introduction

Atrial fibrillation (AF), recognized as the predominant form of clinical arrhythmia encountered globally [1, 2, 3], currently impacts an estimated 35 million individuals. This prevalence is on an upward trajectory, paralleling demographic shifts toward an older population [4]. Achieving and preserving sinus rhythm remains a fundamental objective in managing AF. While pharmacological agents are commonly employed to regulate cardiac rhythm, their application is often limited by adverse effects, particularly in the context of heart failure. Consequently, catheter ablation (CA) has emerged as a vital intervention, especially for those who are either refractory to antiarrhythmic medications or are burdened with heart failure, wherein the safety and tolerability of these drugs are compromised [5, 6].

Owing to their higher resting membrane potential, lower action potential amplitude, smaller maximum phase 0 upstroke velocity, and shorter action potential duration than atrial myocardial cells, myocardial cells located in the pulmonary veins (PVs) become a common ectopic trigger in AF [7]. Consequently, PV isolation (PVI) is generally accepted as the cornerstone of invasive AF treatment. Extensive research has delineated the propensity for anatomic irregularities within PVs to precipitate AF, with such variations manifesting in approximately 18% to 45% of AF cases [8]. Concurrently, investigative efforts have elucidated a broader array of extrapulmonary sites that may act as foci for AF initiation; these include the coronary sinus, left atrial appendage, superior vena cava, and interatrial septum [9, 10]. Furthermore, disparities in left atrial morphology, particularly in proximity to the left atrial crest, have been implicated in AF etiology [11]. The integration of these insights is poised to catalyze advancements in AF ablation methodologies. Prospective developments in electrophysiological modalities promise the advent of tailored ablation strategies, facilitating the pinpointing of idiosyncratic arrhythmic origins and the subsequent selection of bespoke ablation catheters for patient-specific therapeutic interventions.

Current strategies for PVI often involve applying thermal energy or cryotherapy through catheters to induce targeted cardiomyocyte coagulation necrosis, which can easily cause damage to the adjacent atrial tissue, resulting in undesirable complications, such as stroke [12, 13], atrial esophageal fistula [14], phrenic nerve injury [15], coronary artery damage [16], and PV stenosis [17]. Current large-scale clinical surveys and systematic reviews suggest that the complication rate after pulsed field ablation (PFA) ranges from approximately 2% to 5%, reflecting advancements in ablation technology and patient care protocols [18, 19, 20].

PFA is a novel ablation technology that does not use heat or cold energy but involves site-directed intervention using a pulsed electric field (PEF) to make nanoscale hydrophilic micropores appear in the cell membrane of target cells [21], which leads to the expulsion of cellular contents and an imbalance in intracellular homeostasis, eventually inducing programmed cell death. It has strong tissue specificity [22, 23] for cardiomyocytes and is typically applied over a relatively short intervention duration, resulting in necrosis within microseconds [24] for PFA versus seconds for conventional thermal ablation [25, 26]. Moreover, PEF treatment can cause target cell death without destroying the original intercellular connection, thereby ensuring the structural integrity of the atrial tissue after ablation. This review summarizes the detailed mechanisms and research progress in PFA technology, discusses its advantages over traditional ablation technology, and proposes future strategies to address its current limitations.

## 2. Mechanism of PEF Ablation for AF

By killing abnormal pacemakers and conduction cells, catheter ablation (CA) can destroy ectopic pacing sites, interrupt abnormal conduction pathways, and regulate cardiac autonomic innervation to restore the normal sinus rhythm. The main cause of paroxysmal AF (PAF) is believed to be sustained firing in the PV area [27, 28, 29]. Therefore, it is reliable to treat PAF using ablation technology to cause cellular tissue necrosis in the ostium of the PV, that is, by performing PVI. However, the detailed mechanisms underlying persistent AF (PsAF) remain unclear. Since PsAF has been found to have more complicated triggering and maintenance mechanisms, a single PVI is not effective enough to treat PsAF compared to PAF; therefore, additional ablation targets are often needed to improve the efficiency of CA for PsAF.

**Fig. 1.**
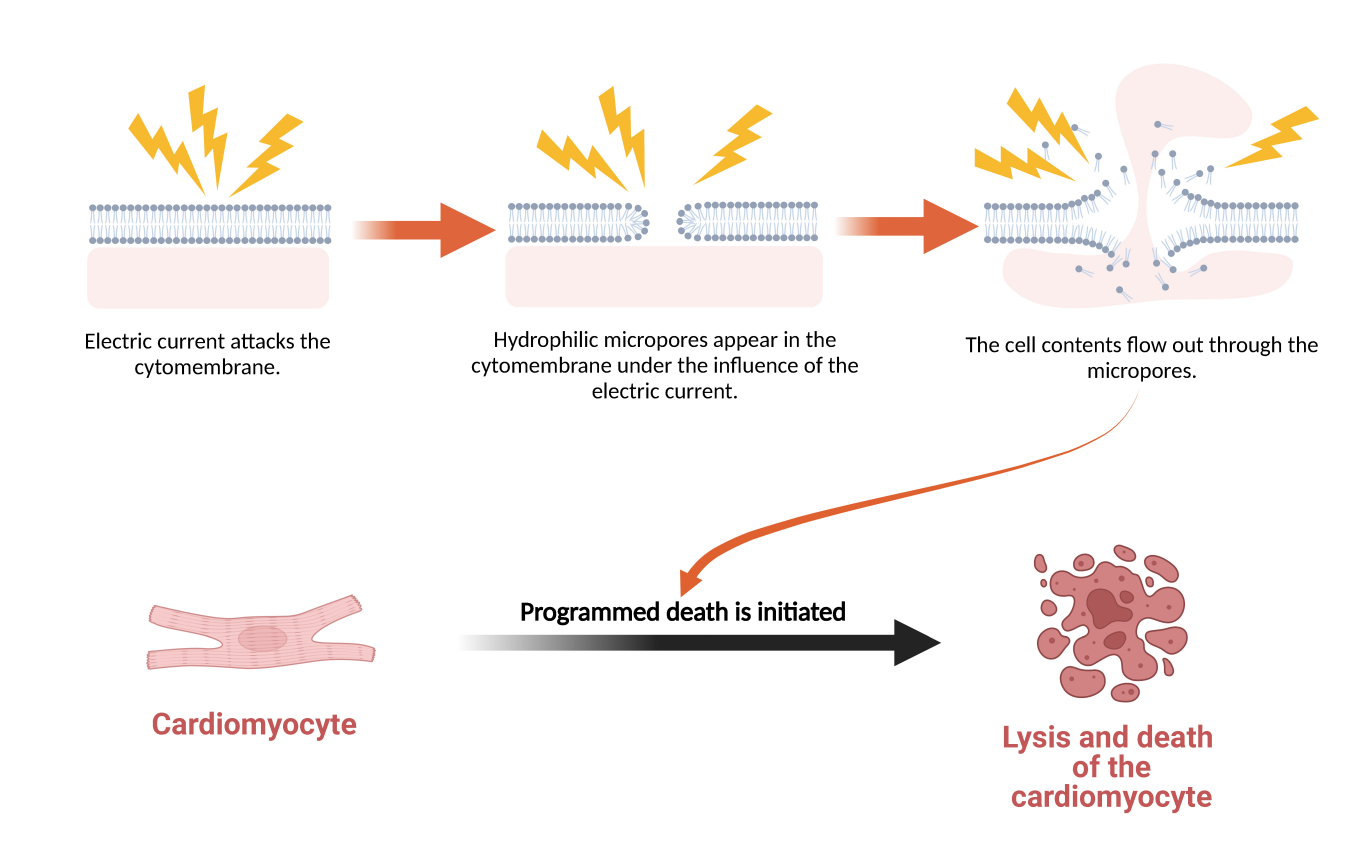
**Mechanism of Cardiomyocyte Death Induced by Electroporation**. Electroporation consists of three stages over time: cell membrane charging, pore generation, and pore radius evolution. When the radius of the hydrophilic pore is large enough, the cellular contents will flow out through the pore, which will disrupt intracellular homeostasis and induce programmed cell death.

PFA is a new technique for AF ablation, which refers to the application of an intermittent high-intensity PEF to a specific type of tissue cell for a very short period (microseconds or nanoseconds), resulting in the electroporation of the cytomembrane. Electroporation can generate hydrophilic pore channels in the lipid bilayer of the cytomembrane, and these membrane channels increase cellular permeability [21]. Increased permeability can be reversible or irreversible, with the former termed reversible electroporation (RE) and the latter termed irreversible electroporation (IRE). There is a stepwise relationship between RE and IRE. IRE is one of the core mechanisms for the necrosis of target cells and tissues during PFA because it causes an irreversible cascade of activated programmed death of targeted cells and tissues [30] (Fig. 1).

With changes in various parameters of the applied PEF, the results of targeted cell tissue intervention may include undetectable electroporation, RE, IRE and thermal damage [31]. Among these, RE and IRE are applied in biotechnology, and thermal damage must be avoided in the construction of irreversible electroporations. A systematic review [32] suggested that a mild hyperthermic state (temperatures between 40 °C and 50 °C) was observed in 30% of IRE-treated regions, and temperatures exceeding 50 °C were observed in 5% of IRE-treated regions, in which the ablation temperature was far lower than that of thermal ablation, sharply reducing the possibility of thermal damage. PFA, while avoiding the thermal injury commonly associated with conventional ablation techniques, is not without its unique set of complications. One such complication, distinct from thermal ablation, is arcing. This phenomenon occurs when intense current density induces gas accumulation at the electrode-tissue interface, escalating impedance and culminating in dielectric breakdown, a sequence that could precipitate myocardial damage [33]. Fortunately, arcing can be avoided by using non-direct current and optimizing catheter electrodes [34].

## 3. Experimental Research Progress of PFA

Prior to the application of PFA in human AF ablation, extensive animal model experiments were performed to investigate its safety and feasibility. This study investigated the degree of myocardial tissue necrosis during endocardial and epicardial PFA, the effects of different PEF voltage intensities and pulses on PVI, and the durability of PVI generated by PEF. Data from previous representative animal experiments are summarized in Table 1 (Ref. [22, 35, 36, 37, 38, 39, 40]).

**Table 1.** **Representative preclinical trials of PFA for AF**.

Experimental subject	Catheter	PEF type and intensity (or voltage)	Ablation position	Acute electrical isolation success	Durable isolation rate	Study Endpoint and follow-up period	Occurrence of complications and creative points	Ref.
Pigs (weight 60–75 kg)	Circular electroporation ablation catheter	Monophasic	Epicardial side of the left ventricle	-	-	3 months	Epicardial PFA caused extensive and deep myocardial necrosis without causing coronary artery injury.	[39]
4-month-old female Yorkshire-mix pigs (70.66 ± 3.5 kg)	A 9-electrode circular array PV ablation catheter	Biphasic, 500 V	Right superior PV ostium, LAA, and RAA	100%	-	-	The replacement of fibrosis by PFA was more uniform than that by RFA. Pathological examination showed that epicardial adipose inflammation was significantly reduced and vascular remodeling was decreased. There were no collateral damages.	[35]
Yorkshire swine (60–70 kg)	Lattice-tip catheter with expandable nitinol mesh with 9 surface microelectrodes and thermocouples	Biphasic	Endocardial: RSPV, SVC, and ICPV	100% (25/25)	61.5% (${\mathrm{PF}}_{\text{LD}}$: 5/6 (83.3%) RSPV, 2/6 (33.3%) SVC, and 1/1 (100%) ICPV)	2 or 4 weeks	Pathological examination showed no damage to mediastinum or pleura. Atrioventricular structural tests after ablation showed no loss of integrity.	[40]
					100% (${\mathrm{PF}}_{\text{HD}}$: 6/6 (100%) RSPV and 6/6 (100%) SVC)			
Yorkshire swine (65–90 kg)	A 7.5Fr bidirectional deflectable catheter with an expandable conductive lattice electrode	Biphasic, 400 V/cm	Endocardial: from SVC to IVC	100% (7/7)	85.7% (6/7)	18–37 days	PEF during ablation was not sufficient to cause damage to phrenic nerve fibers.	[38]
Yorkshire swine	A 7.5Fr circular catheter having 10 electrodes with individual irrigation pores	Biphasic, 1800 V	Endocardial: along the posterior wall from SVC to IVC	100% (12/12)	91.7% (11/12)	30 days	The phrenic nerve and esophagus showed no injury in pathological observation. No obvious bubbles and electric sparks were produced during ablation.	[36]
Swines	A multielectrode circular IRE catheter with 10 ablation electrodes	Biphasic	Endocardial: RIPV, RSPV, and SVC	100%	100% (subchronic), 100% (chronic)	Subchronic (7 ± 3 days); Chronic (30 ± 3 days)	After ablation, myocardial fibers and cardiomyocytes were necrotic, but the structure of myocardial tissue was preserved.	[37]
Female Yorkshire swine (60–70 kg)	Multielectrode pulsed field ablation catheter deployed in flower pose	Monophasic, 800~1800 V and biphasic, 800~1800 V	Endocardial: PV and SVC	Monophasic: 100%; Biphasic: 100%	Monophasic: 55.6% (1/7 SVC, 9/11 PV); Biphasic: 100% (6/6 SVC, 12/12 PV)	10 weeks	No pulmonary vein stenosis was reported. Biphasic PFA had more advantages in durable PVI than monophasic PFA.	[22]

Summary of related core parameters of PEF, effect of electrical isolation, and safety of ablation in animal experiments with PFA. PFA, pulsed field ablation; PEF, pulsed electric field; AF, atrial fibrillation. Isolation success, if without special label, it indicated PVI rate; PVI, pulmonary vein isolation; PV, pulmonary vein; LAA, left atrial appendage; RAA, right atrial appendage; RIPV, right inferior pulmonary vein; RSPV, right superior pulmonary vein; SVC, superior vena cava; ICPV, inferior common pulmonary vein; IVC, inferior vena cava; ${\mathrm{PF}}_{\text{HD}}$, high-dose pulsed field; ${\mathrm{PF}}_{\text{LD}}$, low-dose pulsed field; SPV, superior pulmonary vein; RFA, radiofrequency ablation; IRE, irreversible electroporation; Subchronic, the PFA was evaluated over 7 ± 3 days from ablation; Chronic, the PFA was evaluated over 30 ± 3 days from ablation.

The experimental results shown in Table 1 indicate that the application of PFA for AF is safe in animals. Moreover, compared with thermal energy ablation, the pathological findings suggest that PFA was better able to preserve the structure of the myocardial tissue and cardiac contractility. Furthermore, preclinical studies have shown that biphasic asymmetric pulses can effectively reduce muscle contractions and decrease the threshold for ablation [41]. Additionally, these experiments showed better AF prognosis with PFA compared to traditional AF ablation techniques [42].

Since 2018, several research teams have applied PFA in clinical AF ablation and further optimized the parameters of cardiac PFA in humans. The results of these clinical trials continue to show the superiority of PFA for AF treatment, including a higher success rate of acute PVI, a higher rate of durable PVI, a shorter operation time, and a lower incidence of adverse complications. The representative clinical trials are summarized in Table 2 (Ref. [24, 43, 44, 45, 46, 47, 48]).

**Table 2.** **Representative clinical trials of PFA for AF**.

Experimental subject	Catheter	PEF type and intensity (or voltage)	Ablation position	Acute electrical isolation success	Durable isolation rate	Study endpoint	Follow-up period	Occurrence of complications and creative points	Ref.
Patients with symptomatic paroxysmal AF resistant to at least one antiarrhythmic drug	A pentaspline catheter (flower configuration) containing 5 splines, each with 4 electrodes	Biphasic, 900–2500 V	Endocardial: The ring-shaped area of the LA-PV junction	100% (15/15)	-	-	-	One month after PFA.	[46]
Patients with symptomatic paroxysmal AF resistant to class I to IV antiarrhythmic medications	A pentaspline catheter (flower configuration) containing 5 splines, each with 4 electrodes	Monophasic, 900–1000 V; Biphasic, 1800–2000 V	Endocardial: the ostium of the left superior PV (left) and the right inferior PV (right)	100% (15 in monophasic; 66 in biphasic)	45% (monophasic), 43% (biphasic 1), 56% (biphasic 2), 100% (biphasic 3)	A composite of major safety events	75 days (PEFCAT) or 90 days (IMPULSE) after the index ablation procedure	No recurrence of symptoms and ablation complications were detected after operation. Biphasic electric field did better in PVI.	[24]
Patients with paroxysmal AF resistant to at least one class I to IV antiarrhythmic drug	A pentaspline catheter (basket/flower configuration) containing 5 splines, each with 4 electrodes	Monophasic, 900–1000 V; Early biphasic, 1800–2000 V; Optimized biphasic, 1800–2000 V	Endocardial: cavotricuspid isthmus	100% (57 in monophasic; 223 in early biphasic; 195 in optimized biphasic)	45% (monophasic), 84% (early biphasic), 96% (optimized biphasic) (after 93.0 ± 30.1 days)	Incidence of early and late onset serious adverse events, which were device or procedure related as determined by the independent Clinical Events Committee	30 days, 75 days (PEFCAT and PEFCAT II studies) or 90 days (IMPULSE study), 6 months, and 12 months	No significant complications were detected during the one year follow-up, and only 7 patients had recurrent AF, which was not triggered by PV.	[44]
Patients between 18 and 75 years of age with documented symptomatic persistent AF (AF duration: 7–365 days) refractory or intolerant to at least one Class I/III antiarrhythmic agents	A pentaspline catheter (basket/flower configuration) containing 5 splines, each with 4 electrodes	Biphasic, 1600–2000 V (optimized by previous studies)	Endocardial: cavotricuspid isthmus and LAPW	100% (both PV isolation and LAPW isolation)	PV isolation: 96% (82/85); LAPW isolation: 100% (22/22) (after 76–90 days)	A composite of major safety events	Repeated invasive mapping at 75 days after the index procedure	No significant complications were found during follow-up. PFA had a good and durable effect of electrical isolation in patients with persistent AF.	[47]
Patients with symptomatic paroxysmal or persistent AF	A pentaspline catheter (basket/flower confguration)	Biphasic, 1800–2000 V	Endocardial: cavotricuspid isthmus	100% (137/137)	90.4% (Paroxysmal AF), 60.3% (Persistent AF) (after 1 year)	The primary endpoint was electrical PVI	1 year	No obvious adverse reactions were detected. RIPV may require the PFA to be applied several times to achieve isolation. Atropine reduced the duration of post-PFA asystole and heart block.	[48]
Patients with symptomatic paroxysmal or persistent AF recurrence after first ablation	A pentaspline catheter (basket/flower configuration)	Biphasic, 1800 V; Biphasic, 1900 V; Biphasic, 2000 V	Endocardial: RSPV, RIPV, LSPV, LIPV, and LCPV	100% (25/25)	90.9% (10/11 in the 2000 V group; 64.2% (9/14 in the 1800 V and 1900 V groups)	The rate and distribution of PV reconnection, the features of recurrent atrial tachycardia, and ISAPW (%) after the current pentaspline PFA catheter-guided PVI. ISAPW(%) = (isolated PW surface area)/(total LAPW surface area) × 100	4.8 ± 3.6 months	A single case of postprocedural Dressler’s syndrome was observed 4 weeks after the second ablation. The use of 31 mm catheter was supposed to be associated with a lower reconnection rate. In trend, LSPV was the vein with the most frequent reconnection.	[43]
Patients aged 18–70 years with a diagnosis of paroxysmal AF	A flexible linear epicardial catheter incorporating a guidewire lumen	Biphasic, 900–2500 V	Epicardial: encircling the posterior LA and the 4 PVs	86% (6/7)	-	-	1 month	No adverse events were reported during one month of postoperative follow-up. In the only case where electrical isolation failed, the patient could not be operated on for technical reasons.	[46]
Patients 18–80 years of age undergoing first-time CA of paroxysmal or persistent AF that failed at least one antiarrhythmic drug (class I or III)	A 9-gold circular electrode array	Biphasic, 500–1500 V	Endocardial: near the level of the PV carina	100% (38/38)	-	(1) the inability to isolate all targeted PVs (assessed for entrance and, where assessable, exit block) during the index ablation procedure or (2) ablation using a nonstudy device to isolate any PV	1 month	During 30 days of follow-up, no complications related to the PFA system occurred. Only one serious procedure-related event related to vascular access was reported.	[45]

Summary of relevant core parameters, effects of electrical isolation, and duration, safety, and feasibility of ablation procedures in a PFA clinical trial. AF, atrial fibrillation; PFA, pulsed field ablation; PEF, pulsed electric field; Isolation success, if without special label, indicated PVI rate; PVI, pulmonary vein isolation; PV, pulmonary vein; LA, left atrium; LAPW, left atrium posterior wall; ISAPW%, ratio of the isolated-to total surface area on PW; LSPV, left superior PV; LIPV, left inferior PV; RIPV, right inferior PV; RSPV, right superior PV; LCPV, left common PV; IMPULSE, a safety and feasibility study of the IOWA approach endocardial ablation system to treat atrial fibrillation; PEFCAT, a safety and feasibility study of the FARAPULSE endocardial ablation system to treat paroxysmal atrial fibrillation; PEFCAT II, expanded safety and feasibility study of the FARAPULSE endocardial multi ablation system to treat paroxysmal atrial fibrillation.

As shown in Table 2, PFA PVI has a high success rate in achieving and maintaining sinus rhythm. Acute PVI success rates reached 100% in the experiments, and the rates of sustained PVI post-PFA generally exceeded 90% under optimized biphasic electric fields. Some groups with high rates of electrical reconnection were assumed to have inappropriate parameters. For example, one study [43] suggested that a 31 mm catheter could induce lower reconnection rates than a 35 mm catheter, and the group that utilized an electrical field of 2000 V showed apparently higher durable isolation rates than the group that utilized an electrical field of 1800 V or 1900 V. Contrastingly, in patients with PsAF, the rates of durable PVI post-PFA typically range from 60% to 70%. Recent clinical evaluations of PFA in PsAF cohorts indicate that approximately one-third of patients may experience electrical reconnection following ablation [49, 50, 51], a rate comparable to that observed with traditional thermal ablation techniques [52]. Moreover, patients with PFA had a shorter procedure time and fewer adverse events. It is worth noting that the advantages of biphasic PEF have been clearly reflected in comparative tests [24, 44], and novel multielectrode catheters have been widely used in clinical experiments; however, epicardial ablation may require more experiments to optimize the operating equipment and parameters compared to endocardial ablation.

A pivotal study [53] “Pulsed Field Ablation to Irreversibly Electroporate Tissue and Treat AF” monitored patients with AF for 12 months after PFA and demonstrated a very low incidence (0.7%) of PFA procedure-related adverse events, including no PV stenosis, phrenic nerve damage, or esophageal damage. Furthermore, in 56.1% (95% confidence interval [CI], 46.7–62.7) of patients with PsAF and 66.2% (95% CI, 57.9–73.2) of patients with PAF, PFA was effective at the 1-year follow-up. In the comparative analysis reported by Reddy *et al*. [54], the efficacy of PFA was juxtaposed against that of conventional thermal ablation in a rigorously controlled clinical setting. Of the cohort, 73.3% (204/278) of patients undergoing PFA and 71.3% (194/272) of those subjected to thermal ablation successfully achieved the primary efficacy endpoint, defined as the absence of atrial fibrillation one year after the procedure. These data suggest equivalence in the therapeutic outcomes between PFA and its thermal counterpart, reinforcing the noninferiority of PFA in the context of atrial fibrillation management.

## 4. The Superiority of PFA in Treating AF

### 4.1 Electroporation has Tissue Specificity

PFA is distinguished by its targeted specificity to cardiac myocytes [22], offering a focused therapeutic approach within a concise procedural timeframe. This selectivity is underpinned by empirical studies demonstrating the method’s precision and efficiency [23]. PEF has tissue specificity in inducing IRE; the parameters of PEF can be adjusted to induce IRE in different types of tissue cells, which greatly reduces the incidence of collateral tissue damage in PFA. Table 3 (Ref. [55, 56, 57, 58, 59, 60]) shows the electric field threshold of IRE for different types of tissues under certain pulses. The following is a comprehensive analysis of the effects of PEF on ablation sites and adjacent tissue cells in a series of PFA experiments for AF [55, 56, 57, 58, 59]; when the electric field environment of a certain tissue reaches its corresponding threshold, it undergoes IRE and initiates apoptosis or necrosis.

**Table 3.** **The electric field intensity threshold of irreversible electroporation (IRE) for different types of tissue**.

Tissue Type	Electric field intensity threshold of IRE (V/cm)	Pulses (µs)	Ref.
Myocardium	750	100	[55]
Vascular smooth muscle	1750	100	[56]
Nerve	1000	50	[57]
Liver	805	100	[58]
Kidney	575 ± 67	100	[59]
Pancreas	506 ± 66	70	[60]

#### 4.1.1 Myocardium

According to the mechanism of electroporation, different electric field intensities cause varying electroporation effects, and the sensitivity of certain tissues to electric field intensities varies. This may be due to cell size, cell metabolic activity, and PEF pulses. During atrial fibrillation ablation procedures, the strategic targeting of cardiomyocytes is essential, with the objective of selectively ablating these cells while concurrently conserving the integrity of neighboring structures. These include the vasculature, nerve fibers, adipose tissue, smooth muscle, and mucosal layers within the cardiac milieu and in proximal organs such as the esophagus, which are critical to preserve to minimize collateral damage. Cardiomyocytes are most sensitive to IRE at a certain pulse. An electroporation experiment on cardiomyocytes (H9C2) showed that effective myocardial injury occurred when the intensity of PEF was greater than 375 V/cm [61]. Therefore, PFA has distinct tissue specificity and can preferentially ablate myocardial tissue while protecting other collateral structures (such as the esophagus and phrenic nerve) from injury.

#### 4.1.2 Esophagus

Because the esophagus is adjacent to the posterior part of the left atrium (LA), collateral damage to the esophagus can easily occur during ablation of the left atrial posterior wall (LAPW), leading to atrial esophageal fistula (AEF), which is the most serious complication of CA. In a study [62] of 190 patients with AEF, 80.82% developed AEF within 30 days after ablation, and the overall mortality was 63.16%. Ablation experiments in pig models have shown that the application of PFA through the aorta or inferior vena cava, which is adjacent to the esophagus, does not cause esophageal injury [35, 63]. However, if PFA is applied directly to the esophagus, it can cause transmural cell death around the ablation sites. However, owing to the complete structure and function of the extracellular matrix, it is postulated that the esophagus may possess the capacity for self-repair following PFA [64]. Although PFA is nonthermal, it generates a negligible amount of heat. Fortunately, the histological morphology shows that the thermal lesion of the esophagus is confined to the muscular layer and does not spread to the epithelial and mucosal layers, as radiofrequency ablation (RFA) does [65, 66, 67, 68].

#### 4.1.3 Pulmonary Veins

Most AF is caused by anomalous pacing sites at PVs, and it has been found that durable PVI is associated with a lower rate of AF recurrence after CA [69]; therefore, durable PVI is the key to successful AF ablation. Despite the widespread use of CA in PVI, PV stenosis caused by CA remains a common problem [70]. PFA appears to be a promising method to resolve this issue. In a previous study using a canine model [71], the extent of PV stenosis induced by PFA and RFA was compared using the cross-sectional area after ablation, and the results showed that PFA significantly reduced the risk of PV stenosis. Subsequently, animal experiments have shown that the likelihood of PV stenosis caused by PFA is extremely low [35, 72]. In addition, in the clinical trials mentioned in Table 2, the initial success rate of PVI was 100%, and there were no reported cases of PV stenosis. Kuroki [73] directly reanalyzed 299 PVs from 80 patients with AF and compared the extent of PV stenosis in patients with PAF treated with RFA and PFA in four different trials and found that 9.0% (15 of 166), 1.8% (3 of 166), and 1.2% (2 of 166) of PVs from patients treated with RFA turned out to be mild, moderate, or severe narrowing, respectively, while in patients treated with PFA, no cases of PV narrowing or stenosis were detected.

#### 4.1.4 Coronary Artery

In a systematic review of CA-related coronary injuries, different vascular lesions, including vasospasm-related coronary occlusion, coronary artery dissection, and plaque rupture, occurred near the ablation site [74]. In thermal ablation (cryoballoon ablation and RFA), damage can be minimized by heating or cooling the blood flow to protect the coronary arteries surrounding the ablation sites. For example, some animal experiments have successfully minimized heat-induced damage to the coronary artery endothelium during RFA using intracoronary irrigation with chilled saline [75]. However, when the lesion is too close to blood vessels, this protective strategy fails.

Fortunately, PFA-induced tissue damage is nonthermal and noncontact, which greatly reduces the risk of coronary arterial injury. In a study on coronary artery injury using a porcine model [76], no intimal hyperplasia or signs of stenosis were observed in the group with epicardial IRE on the left anterior descending artery. Meanwhile, in the group with epicardial IRE at the base of the left ventricle, 5 of 56 inside and 1 of 47 outside lesions had intimal hyperplasia but with <50% area stenosis. Furthermore, connective tissue growth factor expression was observed in the scar tissue but not in the fibrotic tissue directly surrounding the arteries, indicating that the arteries were indeed spared from tissue damage and remodeling. Even in the presence of large myocardial lesions, the coronary arteries remained free of clinically relevant damage 3 weeks after epicardial IRE ablation. Another study on swine [77] also suggested that direct epicardial PFA on coronary arteries created myocardial lesions but also caused neointimal hyperplasia, inducing chronic mild stenosis and acute moderate spasm of the coronary arteries. These studies indicated that coronary artery damage caused by PFA is normally induced by intimal hyperplasia and is always mild or moderate, which cannot induce long-term coronary artery dysfunction. In the future, it may be possible to reduce the risk of coronary artery spasms by inhibiting intimal hyperplasia.

During PFA procedures, instances of coronary spasm have been frequently reported, with these spasms typically presenting as reversible and not leading to fatal outcomes [78, 79]. Research suggests that ablation in close proximity to coronary arteries heightens the risk of such spasms, yet they seldom escalate to myocardial infarction [80]. Prophylactic use of nitroglycerin has shown efficacy in the prevention of these spasms [81]. The transient nature of coronary spasms observed with PFA may be due to the procedure’s ability to induce reversible electroporation and permeabilization in both cardiomyocytes and adjacent vascular endothelial cells, which does not result in permanent tissue damage [79].

#### 4.1.5 Phrenic Nerve

Similar to other anatomically adjacent tissues, the phrenic nerve is susceptible to thermally induced damage from CA. IRE has also been shown to cause minor damage to the nerves surrounding the ablation site [82], but animal experiments [83] have shown that only acute paralysis of the phrenic nerve, without evidence of chronic injury, occurs with therapeutic PFA. However, several recent animal experiments and clinical trials have shown that PFA does not lead to clinically significant phrenic nerve injury [36, 45]. A study [84] of 18 patients with AF indicated that serum nerve injury biomarkers did not change preablation, immediately postablation, or 24 h after ablation. Furthermore, a clinical case report [85] including three patients aged 55–81 years who underwent PFA for symptomatic AF indicated that induced phrenic nerve palsy lasted less than 1 min and was followed by spontaneous full recovery in all cases. The pathophysiological mechanism underlying transient phrenic nerve dysfunction has been identified as hyperpolarization of the phrenic nerves. Further research is needed to resolve the connection between IRE and hyperpolarization, which may be related to electroconformational changes in voltage-gated ion channels or ${\mathrm{Ca}}^{2+}$ ion flow induced by the electric field of IRE.

In conclusion, PFA has good tissue specificity and can effectively prevent a series of collateral tissue injury complications associated with traditional thermal ablation. Contrasts drawn from clinical trials [54, 86] reveal that PFA may offer a reduction in operative duration yet necessitate an extended period of fluoroscopy relative to traditional thermal ablation. The incidence of perioperative complications and the rates of AF recurrence within the first postoperative year did not significantly differ between the two modalities. This could be attributed to the nascent stage of proficiency in PFA application. There is a consensus in the literature suggesting the need for more extensive randomized controlled trials with prolonged monitoring to definitively ascertain the comparative long-term effectiveness and safety profiles of PFA versus thermal ablation.

### 4.2 PFA has an Effective and Durable Effect on PVI in Patients with PAF

PFA has obvious advantages in PVI compared to a series of traditional thermal ablation techniques, including RFA and cryoballoon ablation [87]. This advantage is reflected not only in the improved safety and reduced complications of ablation but also in the upfront success rate and durability of PVI, which can have important long-term prognostic implications for AF.

Compared with thermal ablation, PFA showed obvious advantages in single-shot PVI. In a 2022 experimental report [88], the success rate of single-shot PVI induced using PFA in 191 patients with AF was 99.5% (779/783). Comparatively, the isolation rates of single emission and single mapping of cryoballoon ablation and laser balloon ablation were 86% and 91.6%, respectively [89, 90]. Moreover, a different, less supportive guidewire was used in all four instances. Using a modified catheter, all the remaining PVs were isolated after a second series of PFA. Subsequent electrophysiological assessments have elucidated the effects of PFA, demonstrating substantial lesion formation around the pulmonary veins and achieving consistent isolation of the left atrial posterior wall. Notably, this is accomplished with a minimal reduction in tissue voltage, attesting to the precision of PFA [91]. While the incidence is reduced, the phenomenon of early pulmonary vein reconnections post-PFA does manifest. These reconnections tend to localize to specific anatomical regions: the right carina, the anterior segment of the right superior pulmonary vein (RSPV), the posteroinferior quadrant of the right inferior pulmonary vein (RIPV), the posterior sector of the left superior pulmonary vein (LSPV), and the posteroinferior area of the left inferior pulmonary vein (LIPV). Such a distribution mirrors the patterns often observed following thermal ablation procedures. A possible explanation for this similarity may lie in the variable myocardial wall thickness across these regions, coupled with the inherent challenges associated with catheter maneuverability [50, 92].

Over time, owing to incomplete tissue ablation, recovery of the PV electrical connection and recurrence of AF are challenges faced by all types of ablation techniques. Therefore, long-term maintenance of sinus rhythm after PVI is also an important metric for evaluating the relative efficacy of different ablation techniques. In individuals with PAF unresponsive to antiarrhythmic drug therapy, PFA has been administered to achieve sustained PVI [93]. Follow-up investigations, conducted at a median interval of 84 days, revealed cardiac voltage maps consistent with those observed immediately postprocedure in a cohort of 20 patients. This continuity suggests that the ablation-induced isolation of pulmonary vein antral regions may be enduring, hinting at PFA’s capability for establishing long-lasting PVI in PAF patients. In contrast, current data do not demonstrate a comparable advantage for PFA in achieving enduring PVI among patients with PsAF [49, 50, 51].

Although some unresolved issues remain, the feasibility, safety, and durability of PFA for PVI have been demonstrated, and PFA has improved tissue safety compared with conventional thermal ablation. Researchers have made strides in multiple domains to further improve the efficacy and safety of PFA PVI. Currently, a more effective method is the use of a multipolar catheter instead of a traditional single catheter combined with biphasic PEF for ablation [94, 95].

### 4.3 PFA is Ultrarapid and has a Protective Effect on Atrial Structure

The PFA has obvious advantages in terms of ablation speed. In recent clinical trials [24, 44, 46], the operation time of PFA has generally been controlled at approximately 95–25 min. According to a recently published multinational survey [78] of clinical PFA applications, the average procedure time was 65 min (range, 38–215 min), including pre- and/or postablation electroanatomical mapping in some patients. The fluoroscopy time was 13.7 min (range 4.5–33).

PFA has a protective effect on atrial structure. In experimental studies, in addition to mild inflammation in the early postprocedural stage, the atrial site only showed loss of cardiomyocytes and smooth muscle cells, without destruction of the original cardiac tissue structure, replacement with fibrocytes, formation of new blood vessels, or deposition of collagen [37]. This finding indicates that PFA can maintain normal atrial morphology. Studies have shown that during the atrial recovery period after PFA, the physiological process of chronic fibrosis is less involved [96], which can maintain the tissue compliance of the LA and preserve the cardiac structure and contractile and diastolic functions as much as possible.

## 5. Limitations of PFA in the Treatment of AF

### 5.1 Uncertainty of the Ablation Catheter, Electric Field Parameters, and Ablation Positions

The main limitations of the clinical application of AF ablation using PEF are that the selection of an appropriate catheter, optimal parameters of PEF, and ablation positions with high safety have not yet been standardized [64], and these factors can affect the reliability of AF ablation to different degrees. However, with the increasing application of PFA in animal models of AF, these problems will gradually be resolved. In this case, we should analyze the limitations of the current PFA technology in terms of therapeutic effects, which can be used as a reference and research direction for future technical optimization to ensure the efficacy and safety of PFA.

### 5.2 Unknown Characteristics of Cardiac Lesions after PFA

The application of PEF in CA for AF produces unique ablation characteristics in the circumferential venous sinus isolation zone. In 2022, a study [97] described the extent of the PVI zone formed by a single PEF. In this study, 40 patients with PAF or PsAF who had not undergone ablation therapy were treated with PFA (flower/basket-shaped catheter) for the first time. During the 190-day postoperative follow-up, AF recurred in only four patients (15%). High-density three-dimensional electrical mapping was used to compare measurements before and after ablation. Finally, researchers found that an inadequate isolation area was most common in the anterior vena cava segment of the left PV; the greatest area of inadequate isolation in the PV sinus segment was also located anteriorly in the left PV and anteriorly and inferiorly in the right inferior PV. At the same time, an enlarged left atrial isolation zone was most common and widespread in the posterior wall and apical areas on both sides of the LA. In theory, the expanded electrical isolation zone can block the generation and conduction of ectopic triggers more thoroughly but introduces other potential risks. According to prior clinical experience with RFA, an expanded ablation area can result in the following three risks: (1) injury to adjacent tissues, such as the esophagus and phrenic nerve; (2) excessive atrial scarring resulting in loss of systolic function; and (3) separation of normal atrial electrical conduction and formation of a reentrant pathway leading to malignant tachycardia. The first risk can be avoided by adjusting the PEF parameters and improving tissue specificity. However, the second and third are significant risks of additional regional injuries, and special attention should be given to the protection of LAPW myocardium and the roof of the left atrium during PFA PVI.

### 5.3 Microbubbles Produced during Ablation

Gaseous microemboli during cardiac ablation have long been reported. In previous thermal ablation studies, the generation of microbubbles was associated with rapid carbonization, and gas production was associated with tissue injury and necrosis [64]. Although PEF does not use thermal energy to cause necrosis of tissues and cells, microbubbles still occur. Microbubbles generated by PFA can disappear in a short period without obvious physiological effects [98]. Research delineating the safety profile of PFA has produced mixed outcomes regarding cerebrovascular risks. Initial experimental models have suggested that the microbubbles produced during PFA do not precipitate cerebral embolic events [99, 100]. In contrast, data emerging from recent clinical trials indicate the occurrence of asymptomatic cerebral embolisms in a subset of patients following PFA [53, 54, 101]. The potential for such embolisms to occlude critical cerebral vasculature—and thereby precipitate severe neurological sequelae—underscores the imperative for rigorous investigation into prophylactic strategies that might mitigate this risk.

### 5.4 Abnormal Coronary Microvascular Function during PFA

Monophasic PEF ablation causes skeletal muscle contractions and subtle changes in systemic hemodynamics. Some patients with AF have coronary microvascular dysfunction in the absence of obstructive coronary artery disease, which further leads to reduced myocardial perfusion flow and cardiac dysfunction. Perceived AF symptoms cannot often be relieved after undergoing successful ablation [102]. Coronary dysfunction is likely to induce heart failure, which can lead to recurrent arrhythmias, including AF. Therefore, numerous studies have recently adopted biphasic wave PFA to avoid the risk of coronary artery dysfunction [41]. Recent studies have also shown that preoperative nitroglycerin intervention can prevent the occurrence of coronary artery vasospasm [81], which may become a means to reduce the risk of periprocedural myocardial injury during future AF ablations.

### 5.5 Insufficient Clinical Experience with PFA

Thus far, most trials on PFA only had a few samples and were not randomized controlled trials. The durability of PFA PVI and the rate of recurrent AF must be confirmed through long-term event surveillance. Currently, these two data types are relatively scarce and require further research.

Moreover, it is still unclear how PEF affects other cardiac intervention devices, such as artificial valves, cardiac pacemakers, and coronary stents. The acquisition of long-term outcomes from multicenter randomized trials conducted on targeted clinical groups is imperative to validate the efficacy and safety of PFA. An earlier study [103] indicated that metal intracoronary stents in proximity to the ablation device simply “amplify” the vessel-induced distortion of the E-field with tissue between the artery and ablation electrode, probably being moderately heated during this distortion but not damaged thermally. Initial research into PFA for individuals with cardiac implantable electronic devices (CIEDs) indicates a favorable safety profile. Small-scale clinical investigations have reported that PFA does not compromise the functionality or structural integrity of pacemakers and defibrillators. Specifically, in a cohort of six patients, device performance remained stable post-PFA [104]. Similarly, PFA yielded positive arrhythmia control in a patient with an ICD (implantable cardioverter-defibrillator) [105]. Further studies, including a trial with 20 CIED recipients, suggest that avoiding direct contact between the PFA catheter and the implanted device is essential. Adhering to this precaution, PFA appears to be a viable option for diverse CIED types, using catheters sized 31 mm or 35 mm and energy outputs ranging from 1.9 kV to 2.0 V [106].

The current landscape of PFA for the management of AF shows promise; however, it is imperative to acknowledge that the long-term efficacy and safety profile of this modality remain to be comprehensively characterized. The paucity of extended follow-up data from large-scale, randomized controlled trials poses a notable limitation to the robust evaluation of PFA. This gap in evidence may have consequential implications for clinical decision-making, potentially hindering the development of standardized protocols and the optimization of patient outcomes. This underscores the exigent need for multicenter studies with longitudinal monitoring to substantiate the long-term therapeutic value of PFA and to affirm its role in the evolving paradigm of AF treatment. Such investigations are crucial not only for validating the preliminary positive outcomes but also for ensuring that the benefits of PFA outweigh any delayed adverse effects, thereby enabling informed clinical judgments and enhancing patient care.

## 6. Direction for Technical Optimization of PFA for Clinical AF Treatment

**Fig. 2.**
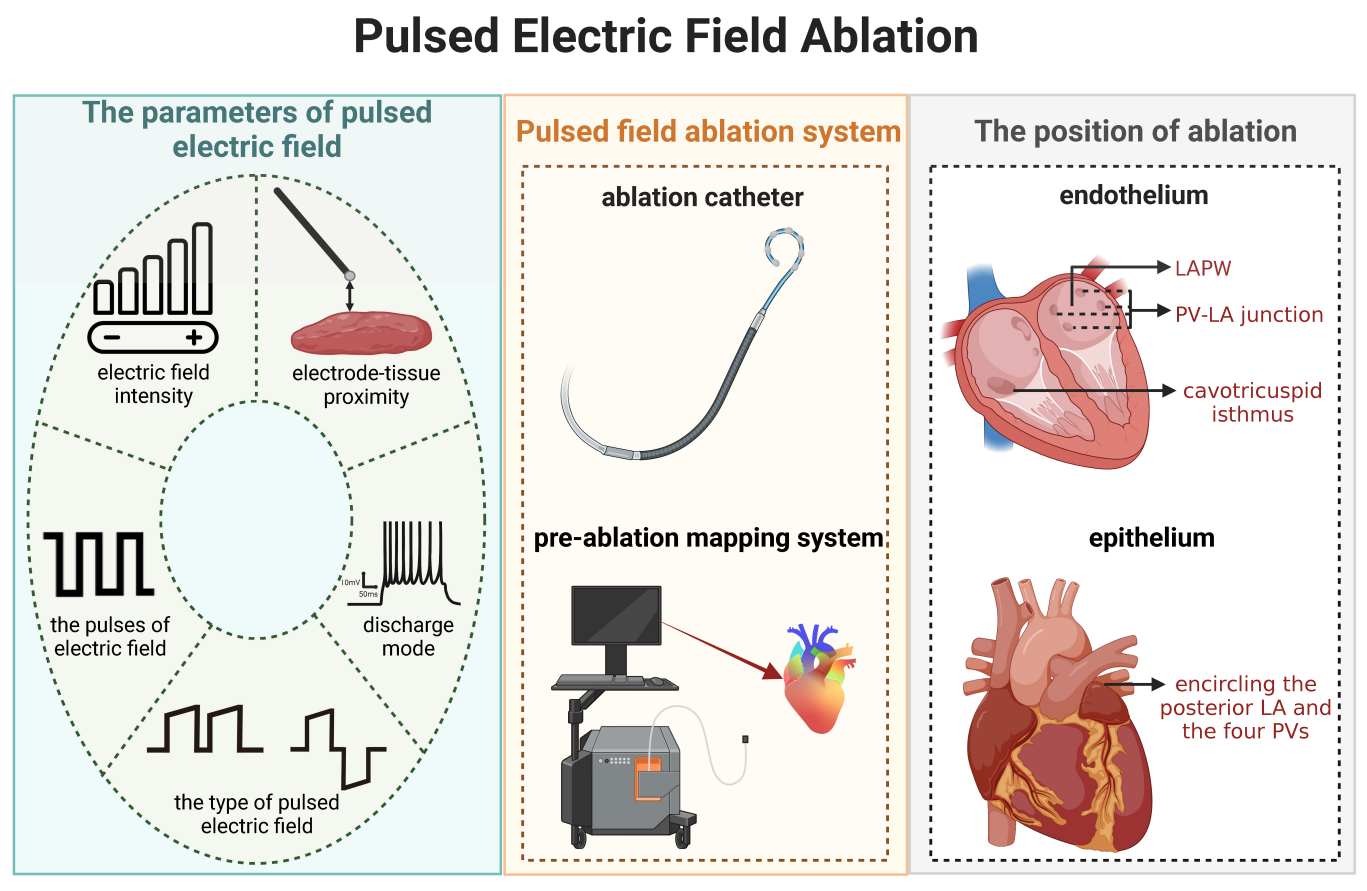
**Future Directions for Optimizing Pulsed Field Ablation-Associated Parameters**. LA, left atrium; PV, pulmonary vein; LAPW, left atrium posterior wall.

According to Joule’s law of electric current, PFA produces not only electrical effects on tissues and cells but also certain thermal effects during the procedure, which can cause thermally induced damage to cells and tissues [107, 108]. At present, the main method for reducing thermally induced damage during ablation is the use of low-frequency PEF. However, according to the mechanism of PFA mentioned above, the formation of IRE depends on PEF parameters, including the electric field frequency. Therefore, one of the main directions for the optimization of PFA technology is to select appropriate PEF parameters to avoid thermal damage and other adverse complications during IRE. Furthermore, the optimization of PEF parameters can partially resolve the technical limitations discussed earlier. However, because of the unclear mechanism of PsAF, the current treatment effect of PVI alone for PsAF is not ideal; therefore, it is necessary to study the efficacy of targeting additional ablation targets to improve the long-term maintenance of sinus rhythm. In addition to optimizing the parameters related to PFA, improving the application of PEF in clinical AF ablation is an important future direction. Fig. 2 summarizes the potential directions for further optimization of PFA technology.

### 6.1 Optimization of the Ablation System Parameters

In the study of PFA applied for PVI, there are many indications that multipolar CA is superior to single CA, even if the parameters of PFA need to be adjusted. Various types of novel multipolar catheters are available. Currently, flower-shaped and basket-shaped catheters are the most common [44, 46]. Special lattice electrodes [26, 38, 109], circular multipolar catheters [36], and flexible linear epicardial catheters [46] have also been used in some experiments. Table 4 (Ref. [24, 44, 45, 46, 109]) illustrates several novel forms of multipole catheters and their performance in the respective experiments. However, an experiment [110] showed that different discharge modes of multipolar catheters would affect the distribution of the myocardial electric field, resulting in different widths and depths of myocardial lesions, which can influence the ultimate efficacy of PVI. Therefore, an important consideration for further optimization of PFA technology is to achieve the needed depth of the lesion while controlling the area of the myocardial lesion by adjusting the discharge mode of the electrodes as part of the multipolar electric field. In addition to improving the catheter form, optimization of the catheter mapping system is also crucial [111]. PEF has the capability to form cardiac lesions without necessitating direct electrode-tissue contact. However, the proximity of the electrode to myocardial tissue significantly influences lesion size, with closer contact resulting in larger lesions. Notably, the variance in lesion dimensions relative to electrode distance is less pronounced with biphasic PEF compared to monophasic PEF [112]. A robust linear relationship exists between lesion depth and electrode-tissue proximity, achieving maximal lesion depth at zero distance [113]. Incorporating a precise mapping system can streamline the ablation process, enabling accurate localization of arrhythmogenic foci and facilitating control over lesion extent through careful management of electrode proximity.

**Table 4.** **Novel catheters in PFA and related respective trials**.

	Flower/Basket-shaped multielectrode catheter	Circular multielectrode catheter	Lattice electrode catheter	Flexible linear epicardial catheter
Catheter conceptual image	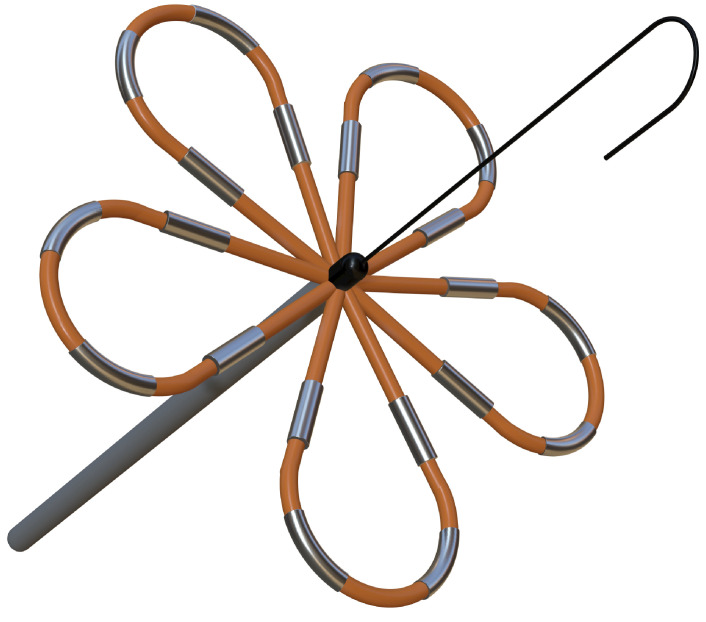 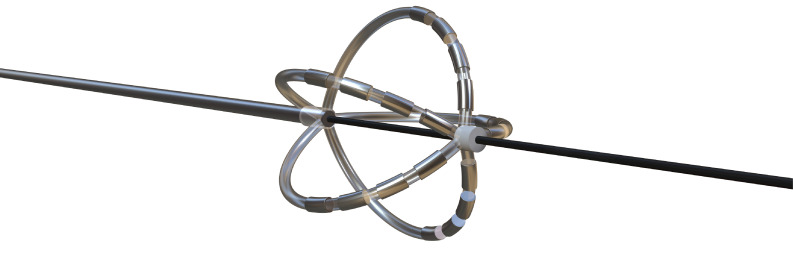	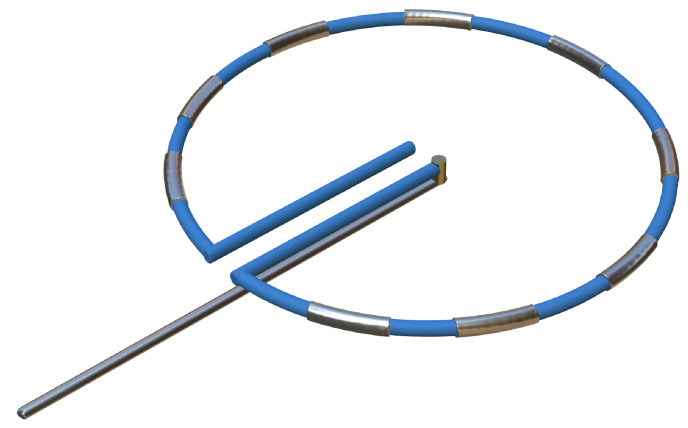	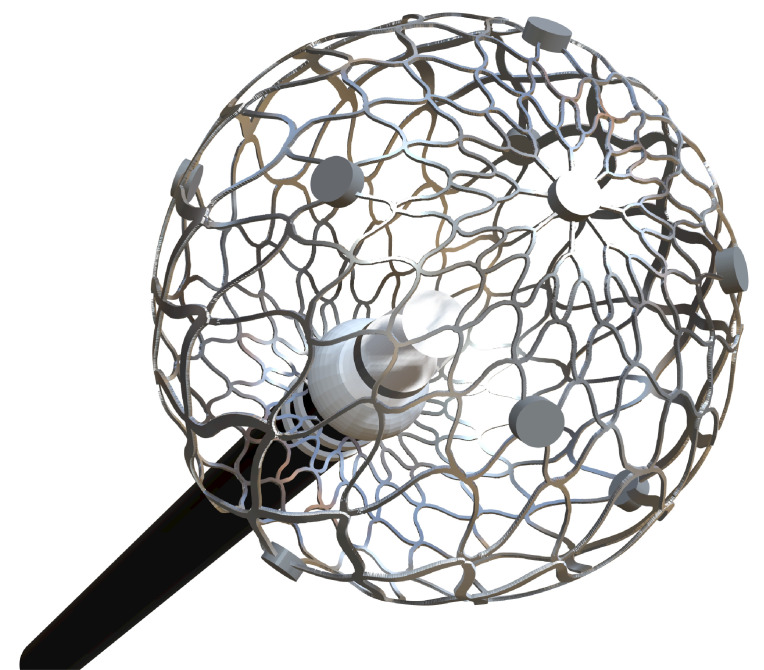	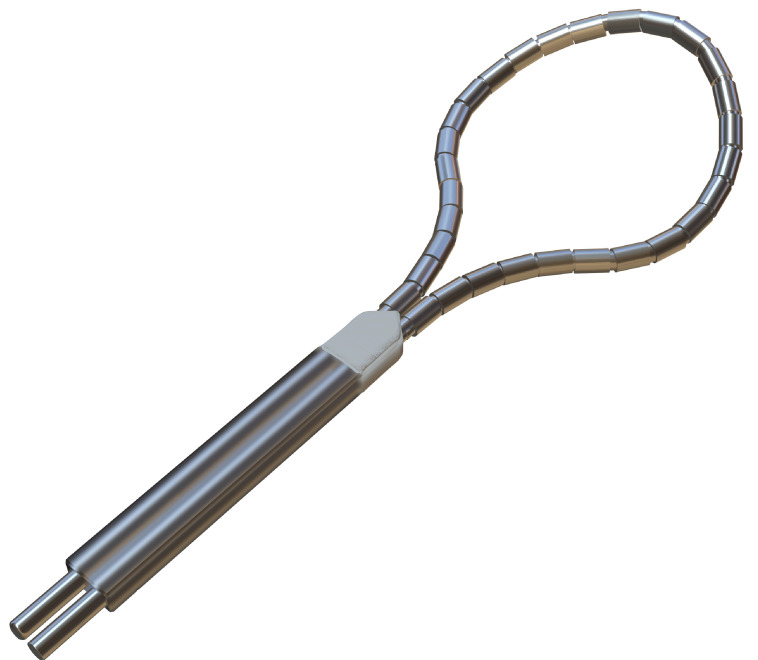
Catheter description	The 12-F over-the-wire PFA ablation catheter has 5 splines that each contain 4 electrodes, and it can be deployed in either a flower petal or basket configuration. When fully deployed into a flower pose, the diameter of the distal portion is 31 mm	An over-the-wire, circular array catheter with 9 gold electrodes (electrode length, 3 mm; 20° forward tilted array with a diameter of 25 mm; 9F shaft)	The lattice catheter is a 7.5Fr bidirectional deflectable catheter with an expandable conductive lattice electrode, containing 9 mini-electrodes/temperature sensors (0.7 mm diameter) that are uniformly distributed on its surface (The catheter is inserted into the sheath in a collapsed form, but once in the heart, the lattice expands to a 9 mm diameter spherical configuration)	This is a flexible linear epicardial catheter and incorporates a guidewire lumen. 30 Electrodes for PEF ablation energy delivery are distributed in the midportion of the catheter—the portion that wraps around the PVs and posterior LA
Experimental subject	Patients with symptomatic paroxysmal AF resistant to class I to IV antiarrhythmic medications	Patients 18–80 years of age undergoing first-time CA of paroxysmal or persistent AF that failed at least one antiarrhythmic drug (class I or III)	Yorkshire swine (65–90 kg)	Patients with symptomatic paroxysmal AF refractory to or intolerant of at least one antiarrhythmic drug
Ablation position	Endocardial: the ostium of the right inferior PV	Endocardial: near the level of the PV carina	Endocardial: from SVC to IVC	Epicardial: encircling the posterior LA and the four PVs
Preablation mapping technique	Preprocedure CT or intracardiac echocardiography (ICE) (Acunav, Siemens, Munich, Germany)	Fluoroscopy or intracardiac echocardiography imaging	Fluoroscopy imaging by lattice electrode catheter	Compatible electroanatomical mapping system (Orion and Rhythmia, Boston Scientific, St. Paul, MN, USA)
PEF type	Biphasic	Biphasic	Biphasic	Biphasic
Electric field intensity or voltage	1800–2000 V	500–1500 V	400 V/cm	2100–2400 V
Results	There were no postoperative adverse events. The acute PVI rate was 100%, and the durable PVI rate under optimized waveforms was 96%	The acute PVI rate was 100%. No adverse events occurred 30 days after the operation. The ablation time was significantly shorter than that of RFA	There was little damage to phrenic nerve and esophagus while durable PVI was formed	There was no PV stenosis, arrhythmia, or pericardial effusion after ablation. The acute PVI rate was 100%
Ref.	[24, 44]	[45]	[109]	[46]

Summary of novel PFA catheters and representative experiments. LA, left atrium; PFA, pulsed field ablation; PEF, pulsed electric field; AF, atrial fibrillation; CA, catheter ablation; PV, pulmonary vein; PVI, pulmonary vein isolation; SVC, superior vena cava; IVC, inferior vena cava; CT, computed tomography; RFA, radiofrequency ablation.

Meanwhile, although the current research on PFA does not involve the study of electrode-tissue orientation, previous ablation operations in various fields seem to default to placing the electrode parallel to the tissue, which may be difficult to achieve accurately in some complex organ locations. However, in the study of parameters affecting IRE, electrode-tissue orientation was found to affect the scope and depth of tissue necrosis and the voltage threshold of IRE [114]. This finding is very important for the optimization of parameters in the ablation process.

### 6.2 Optimization of the PEF Parameters

The meticulous calibration of PEF parameters is integral to achieving ablation specificity, emphasizing the importance of establishing thresholds for the myocardium and surrounding tissues to propel PFA advancements for AF. Optimal thresholds—those precipitating over 80% reduction in cell viability—have been identified, with cardiomyocytes and myocardial fibroblasts exhibiting susceptibility to cell death at an electric field intensity of 1000 V/cm and a pulse quantity of 50 [57].

Moreover, the literature indicates a preference for biphasic short pulses in PFA PVI due to their enhanced efficacy and reduced propensity for muscle contraction, thus preserving atrial architecture post-ablation. Notably, achieving a comparable PVI effect with biphasic short pulses necessitates voltages surpassing 1000 V/cm, unlike monophasic long PEF, which bears implications for potential thermal damage [115]. This necessitates precise PEF parameterization to balance the therapeutic benefits with safety concerns.

Meanwhile, Howard *et al*. [113] showed that the proximity between the electrode and tissue could affect the depth and offset width of the tissue lesion when using a biphasic electric field for ablation. The depth and offset width of the tissue lesion were linearly related to the electrode-tissue proximity; that is, the greater the distance between the electrode and tissue, the smaller the depth and offset width of the necrosis. When the distance was zero, the depth and offset width of necrosis was the maximum. Therefore, while adjusting the PEF parameters, it is possible to control the degree of myocardial necrosis by adjusting the proximity between the electrode and the tissue. The closer the electrode and the tissue, the higher the possibility of achieving myocardial transmural necrosis, while at the same time, the offset width of the necrotic area will increase.

Moreover, in the treatment of AF, the anisotropic electrical conductivity caused by fiber orientation cannot be ignored. According to one study [116], there is a significant difference in the size of the surface ablation area, ablation isosurface, and ablation volume between anisotropic and isotropic electrical conductivity. Consequently, to develop a more targeted restricted ablation zone, it is necessary to establish an electrically refined cardiac model with anisotropic and isotropic electrical conductivities.

### 6.3 Determination of Optimal AF Ablation Positions

The effect of PFA on PVI in the treatment of PAF is widely recognized. PVs play a crucial role in the initiation and maintenance of AF, particularly PAF [117]. However, because of the complexity of the mechanism of PsAF, simple PVI often cannot achieve a sustained therapeutic effect; therefore, it is still necessary to find new ablation targets to improve the prognostic effect of PsAF ablation. PFA exhibits more limited tissue penetration than conventional thermal ablation, necessitating the identification of supplemental ablation targets. While thermal ablation for AF is effective due in part to its impact on the autonomic nervous system by targeting ganglionated plexi (GP), PFA may not achieve the same extent of intramyocardial autonomic nerve ablation, calling into question its thoroughness and persistence [118, 119].

Many studies have shown that LAPW can be used as a reliable new target for the ablation of PsAF [120, 121]. In an earlier study that included LAPW as a target for PFA, 21 patients undergoing ablation did not have serious complications; the durable isolation rate of LAPW was 100%, the durable PVI rate was 96%, and no recurrence of AF was reported during the 76- to 90-day follow-up [47]. Reliable clinical experiments have also shown that the application of LAPW ablation in the treatment of PsAF does not damage the systolic function of LA [122] and has high feasibility and safety. Concerning PFA’s efficacy, achieving comprehensive electrical isolation of LAPW can be challenging. Although recent small-scale trials have shown promising control of arrhythmias post-LAPW PFA [123], the long-term effectiveness and durability of these outcomes warrant confirmation through larger, randomized studies.

In addition to LAPW, another investigator focused on the Marshall bundle and developed the Marshall–Plan ablation protocol [124]. In this protocol, the left atrial sites were targeted sequentially as follows: the coronary sinus and vein of the Marshall musculature, PVI, and anatomical isthmus (mitral, roof, and cavotricuspid isthmus). The patients recruited in this study had a history of long-term PsAF, and the results showed that the maintenance rate of sinus rhythm 12 months after a single ablation was >70% without the use of antiarrhythmic drugs, indicating the high feasibility of this new ablation protocol for the treatment of PsAF. Mitral isthmus PFA has emerged as a viable approach for managing PsAF [80] and is particularly beneficial in cases involving left atrial reentrant tachycardia. However, the applicability of multipolar PFA catheters for mitral isthmus and anterior line ablation may be limited. Focal PFA catheters have been shown to be potentially more suited for these specific ablation targets [125].

The key to finding new ablation targets lies in an in-depth understanding of the electrophysiological mechanism of PsAF. It is now believed that the occurrence and perpetuation of PsAF result from the existence of multiple focal triggers and reentrant pathways in the atrium. Studies have suggested the presence of persistent abnormal activation lesions in the LAPW and three intermittent activation lesions located in the right posterior wall of the atrium. The abnormal conduction pathway had five breaches, two in the PVs, one in the top region of the right atrium, and two in the free wall of the LA [126]. All of these positions may be potential novel ablation targets for AF. Furthermore, it has been found that the formation of an epithelial adipose tissue inflammatory microenvironment, fibrosis, infiltration of atrial tissue, autonomic dysfunction, and oxidative stress are crucial mechanisms that trigger and maintain AF [127]; thus, epithelial adipose tissue may be a novel target for the clinical treatment of AF.

### 6.4 Mode Optimization of PEF Application in Ablation

#### 6.4.1 Combination of Thermal Ablation and PFA

In view of the limitations of the current single-energy source CA, researchers have innovatively combined different modalities to compensate for each other’s shortcomings and achieve better therapeutic efficacy. One study [26] combined RFA and PFA and alternately used these two forms of energy for ablation during catheter intervention. A novel lattice tip ablation catheter with a compressible 9 mm Nitinol tip was used in the experiment, which could perform focal RFA or PFA damage within 2–5 s. This innovative hybrid ablation technique showed a success rate of PVI similar to that of the traditional single form of ablation. Another study [128] combined PFA with ultralow temperature cryoablation, which induced an extended lesion depth beyond cryoablation without causing muscle contractions or microbubbles.

By combining the tissue safety of PFA and the rich clinical success of thermal ablation, the safety of the procedure is also highly guaranteed, but its clinical application requires further exploration.

#### 6.4.2 Epicardial PFA

Traditional ablation positions are typically located in the endocardium. To reduce invasive injury to patients and avoid vascular embolization caused by endovascular operations, researchers attempted epicardial ablation during the era of thermal ablation. Nevertheless, because of the thick fat and muscle layers extending from the epicardium to the endocardium, the experimental results were not satisfactory. Moreover, thermal ablation of the epicardium is more likely to damage the tissue structures near the atrium [129]. Notably, later studies on PsAF found that local atrial abnormal excitation waves could break through the atrial wall, penetrate to the epicardium, and spread from the focal point of the epicardium in all directions, leading to electrical separation of the endocardium and epicardium, resulting in a higher incidence of PsAF [130, 131, 132]. Therefore, there is an urgent need to develop a feasible and safe technique for the ablation of ganglionated plexi embedded within epicardial fat to block abnormal electrical conduction in the epicardium.

The advent of PFA seems to provide a solution to this problem because IRE induced by PEF is tissue specific; therefore, it can be applied for epicardial ablation without fear of damage to the surrounding tissues. Initial experiments with epicardial GP ablation via radiofrequency in canine models revealed an augmented risk for atrial arrhythmias post-procedure, a phenomenon attributed to potential imbalances in autonomic reinnervation. This finding raises the consideration of similar arrhythmic vulnerabilities potentially manifesting after epicardial PFA [133]. A study [134] in a pig model showed that epicardial ablation under PEF produced good transmural myocardial damage without adverse events. Subsequent clinical investigations have corroborated the procedural safety and practicability of epicardial PFA for GP modulation. These studies demonstrate the technique’s ability to alter cardiac autonomic nervous system dynamics during surgical interventions without significant complications [46, 135]. Furthermore, according to computer modeling of epicardial PFA [136], the PEF zone was almost entirely circumscribed by the epicardial fat layer, while the myocardial incidence was extremely low. Full torso and limited-domain computer models for epicardial PFA [137] indicate that the electrical field is mainly limited to the target site (PEF zone with lengths of 25.79–29.00 mm, depths of 5.98–7.02 mm, and maximum widths of 8.75–10.57 mm) and is practically negligible in adjacent organs (<30 V/cm and <36 V/cm in the esophagus and lungs, respectively), with the 400 V/cm isoline being used to estimate the “PEF zone”. While preliminary data affirm the safety of epicardial PFA for ANS (autonomic nervous system) modulation in GP ablation, the true impact on cardiac autonomic function and its efficacy in reducing atrial fibrillation incidence remain to be conclusively determined. Comprehensive, randomized studies are imperative to ascertain the clinical significance of this intervention. Moreover, the incidence of arrhythmias subsequent to certain epicardial ablation procedures necessitates careful consideration [133, 135].

## 7. Conclusions

PFA is an emerging ablation technology for AF with excellent potential. Numerous studies have shown that PFA has an equivalent effect compared with thermal ablation on the establishment of durable PVI and can reduce the occurrence of PV stenosis and collateral damage during both PAF and PsAF ablation procedures. Furthermore, PEF intervention can better protect the atrial structure, avoid extensive damage to the atrial wall, and thereby enhance cardiac reserve. As more experimental and clinical studies are conducted, the existing technical parameters of PFA can be further optimized.

Given the aging global population, which in turn leads to a higher incidence of AF and associated cardiovascular comorbidities, additional research to better address the current limitations, unresolved issues, and unanswered questions associated with PFA is crucial to maximize the potential of this revolutionary technology for the treatment of AF. It is important for future research to construct an electrically refined cardiac model, optimize the discharge mode, establish a novel ablation mode, and observe PFA in a specific population.

## Abbreviations

CA, catheter ablation; IRE, irreversible electroporation; LA, left atrium; LAPW, left atrium posterior wall; PAF, paroxysmal atrial fibrillation; PEF, pulsed electric field; PFA, pulsed field ablation; PsAF, persistent atrial fibrillation; PVI, pulmonary vein isolation; PVs, pulmonary veins; RE, reversible electroporation; RFA, radiofrequency ablation.
